# Association of plasma cystatin C with all-cause and cause-specific mortality among middle-aged and elderly individuals: a prospective community-based cohort study

**DOI:** 10.1038/s41598-022-24722-4

**Published:** 2022-12-23

**Authors:** Jinhua Wu, Yuemei Liang, Rong Chen, Linli Xu, Zejin Ou, Haiying Liang, Lina Zhao

**Affiliations:** 1 Department of Obstetrics, Guangdong Women and Children Hospital, 521 Xingnan Road, Panyu District, Guangzhou, 511442 China; 2 Department of Central Laboratory, Guangzhou Twelfth People’s Hospital, Guangzhou, China

**Keywords:** Diseases, Medical research, Molecular medicine, Risk factors

## Abstract

We investigated the associations of plasma cystatin C with all-cause and cause-specific mortality risk and identified potential modifying factors affecting these associations in middle-aged and elderly people (≥ 50 years). This community-based prospective cohort study included 13,913 individuals aged ≥ 50 years from the Health and Retirement Study. Cox proportional hazard models were used to estimate the associations between cystatin C concentrations and the risk of all-cause and cardiovascular and cancer mortality after adjustment for sociodemographic characteristics, lifestyle factors, self-reported medical history, and other potential confounding factors. During a total of 71,988 person-years of follow-up (median: 5.8 years; interquartile range 3.3–7.6 years), 1893 all-cause deaths were documented, including 714 cardiovascular-related and 406 cancer-related deaths. The comparisons of the groups with the highest (quartile 4) and lowest (quartile 1) cystatin C concentrations revealed that the adjusted hazard ratios and 95% confidence intervals were 1.92 (1.62–2.28) for all-cause mortality, 1.98 (1.48–2.65) for cardiovascular mortality, and 1.62 (1.13–2.32) for cancer mortality. The associations of cystatin C concentrations with all-cause, cardiovascular and cancer mortality did not differ substantially when participants were stratified by sex, age, BMI, current smoking status, current alcohol consumption, and regular exercise (all *P* for interactions > 0.05). Our study indicates that an elevated plasma cystatin C concentration is associated with an increased risk of all-cause, cardiovascular and cancer mortality both men and women among the middle-aged and elderly individuals.

## Introduction

Chronic kidney disease (CKD)^1^ and mild renal dysfunction are associated with an increased risk of mortality among middle-aged and elderly persons^2,3^. Renal function is usually determined by the estimated glomerular filtration rate (eGFR), serum creatinine, blood urine nitrogen, or calculated creatinine clearance. The GFR is considered the best global index of renal function, but direct measurement of the GFR is complex. Creatinine has been applied to estimate the GFR. However, the eGFR is insensitive for the detection of moderate renal dysfunction and is greatly influenced by factors^4–6^, such as age, sex, and muscle mass. Therefore, the early detection of renal-related biomarkers for mild-to-moderate renal injury with an appropriate intervention is likely to promote health and increase life expectancy.

Cystatin C, which is a cysteine protease inhibitor produced by nucleated cells^7^, is considered to be superior to creatinine as a measure of renal function^8,9^. Numerous studies have investigated the association of cystatin C levels with the risk of all-cause and cardiovascular mortality^8,10–12^. For instance, elevated cystatin C concentrations were suggested to be associated with a higher risk of all-cause mortality in many^8,10^ but not all studies^13^. CKD has also been shown to contribute to cancer morbidity and mortality^14–16^. Nonetheless, evidence regarding the relationship between cystatin C concentration and cancer mortality is very limited^17,18^. In addition, whether the associations of cystatin C concentrations with mortality vary by potential modifying factors, such as gender and age group, remain controversial^13,19,20^, which may have important implications for the application of cystatin C.

In the current study, using nationally representative community-based cohort data from the Health and Retirement Study (HRS), we aimed to examine the associations of cystatin C concentrations with all-cause and cause-specific mortality and to identify potential modifying factors affecting these associations among middle-aged and older individuals (≥ 50 years).

## Methods

### Design, study setting, and participants

These study data were obtained from the HRS, which is a nationally representative community-based prospective cohort study of middle-aged and elderly Americans. Details regarding the study design and participants have been previously reported^21,22^. Briefly, the participants were surveyed biennially beginning in 1992; five additional waves of participants were added in phases between 1994 and 2014. Starting in 2006, an enhanced face-to-face survey that included biomarker assessments was implemented in the HRS (http://hrsonline.isr.umich.edu). For this study, we included participants aged ≥ 50 years between 2006 and 2014. Participants with missing cystatin C concentration data or cancer at baseline were excluded. In total, 13,913 participants (5841 men and 8073 women) were eligible. A flowchart of participant enrollment is shown in Figure S1. Ethical approval for the HRS was granted by the University of Michigan Institutional Review Board, who confirms that all experiments were performed in accordance with relevant guidelines and regulations, and all participants provided written informed consent.

### Measurement of cystatin C concentrations

Blood samples were obtained by pricking a participant’s finger with a sterile lancet after cleansing the finger with an alcohol swab^3^. The measurement of cystatin C concentrations was performed with a standard ELISA procedure at the University of Vermont in 2008 and University of Washington in 2012 was assayed from dried blood spots^3^. To account for dried blood spots assay and laboratory variability in cystatin C values, HRS data are released with National Health and Nutrition Examination Survey equivalent assay values^23^, which we used for our analyses. Cystatin C concentrations were classified as belonging to quartile 1 (Q1, < 0.84 mg/L), quartile 2 (Q2, 0.84–0.97 mg/L), quartile 3 (Q3, 0.98–1.20 mg/L), or quartile 4 (Q4, > 1.20 mg/L).

### Assessment of deaths

Deaths were measured in each cohort through data from the National Death Index (NDI) and by exit interviews with family members. Previous HRS analyses indicated a rate of death validation of ~ 99%^22^. Causes of death were categorized according to the International Classification of Diseases 10 (ICD 10). ICD codes I00-I99 and C00-C97 were categorized as cardiovascular mortality and cancer mortality, respectively^24^. We calculated the follow-up time from the baseline survey until the date of death or December 31, 2014, whichever occurred first.

### Covariates

Potential confounding factors included in this study were selected based on previous studies^10,13,25^. The following covariates were included: sociodemographic factors (age, sex, educational level, ethnicity, and household income); lifestyle factors (regular exercise, current smoking status, alcohol consumption, and body mass index [BMI]); laboratory measures (high-density lipoprotein cholesterol [HDL-C], total cholesterol [TC], high-sensitivity C-reactive protein (hsCRP), and hemoglobin A1c [HbA1c] levels); the 8-question Center for Epidemiologic Studies Depression Scale (CES-D 8) score; self-reported prevalent health conditions(heart disease, stroke, hypertension, diabetes, pulmonary disorders, and psychological problems); and limitations in any of the following 5 activities of daily living (ADLs): getting in and out of bed, bathing, walking across a room, dressing, and eating. We classified current alcohol consumption as drinking (one or more drinks per day) or not drinking. BMI was calculated by weight in kilograms (kg) divided by the square of height in meters (m^2^). All covariate data were obtained from the structured questionnaire and biochemical tests conducted at baseline (available at the HRS website: http://hrsonline.isr.umich.edu).

### Statistical analysis

To correct for missing values and reduce the potential for inferential bias, we imputed missing data for the covariates using multiple imputation methods^26^. Baseline tables were generated using descriptive statistics (the mean and standard deviation [SD] or the number and percentage [%]) stratified by cystatin C quartiles. Kaplan–Meier curves were generated for the cystatin C quartiles, and log-rank tests were conducted to compare different groups. Cox proportional hazards models were performed to estimate hazard ratios (HRs) with 95% confidence intervals (95% CIs) for mortality according to the cystatin C quartiles (with the lowest quartile [Q1] as the reference group). We also evaluated the HRs of mortality per 1 mg/L increase in the cystatin C concentration. The Cox proportional hazards assumptions were assessed with Schoenfeld residual plots^27^, and no violation of the assumptions was observed. The baseline model (Model 1) tested the association between cystatin C concentrations and mortality and was adjusted for age and sex, while the multivariable-adjusted model (Model 2) further controlled for the following: ethnicity (white, black, or other); household income (≤ 20,000, 20,001–50,000, or > 50,000 dollars); education level (< 12, 12–15, or > 15 years); BMI (continuous variable); regular exercise (yes or no); smoking status (current smoker or nonsmoker); alcohol consumption (current drinker or nondrinker); HDL-C level (continuous variable); TC level (continuous variable); hsCRP level (continuous variable); HbA1c level (continuous variable); prevalent health conditions (heart disease; stroke; hypertension; diabetes; pulmonary disorders; psychological problems); CES-D 8 score (continuous variable); and limitations in ADLs (yes or no).

Effect modifications of the associations between each 1 mg/L increase in cystatin C concentration and mortality by sex (men or women), age (< 65 or ≥ 65 years), current smoking status (smoker or nonsmoker), current alcohol consumption (drinker or nondrinker), BMI (obese [> 30 kg/m^2^] or nonobese [≤ 30 kg/m^2^]) and regular exercise (yes or no) were measured by computing likelihood ratios. To determine the robustness of the results, we performed sensitivity analyses, including (1) excluding participants who died during the first 2 years of follow-up, (2) stratification by tertiles and quintiles of cystatin C concentration (3) additionally adjusted for measurement of cystatin C concentrations laboratory (the University of Vermont and University of Washington). All analyses were conducted with R software version 4.0.5 (R Foundation for Statistical Computing, Vienna, Austria), and a two-tailed *P* value < 0.05 was considered statistically significant.

### Institutional review board and Informed consent

Ethical approval for the HRS was granted by the University of Michigan Institutional Review Board. Informed consent was obtained from all participants involved in the study.

## Results

Table 1 presents the characteristics of the study participants stratified by quartiles of cystatin C concentrations at baseline. The mean age of the included participants was 65.0 years, and 58.0% (8073) of the participants were women. Compared with participants with lower quartiles of cystatin C concentrations, those with higher cystatin C concentrations were more likely to be white, less educated, and less frequent drinkers; those with higher cystatin C concentrations were also more likely to have a higher BMI. The prevalence rates of heart disease, stroke, psychological problems, pulmonary disorders, hypertension, diabetes, and limitations in ADLs increased with increasing quartiles of cystatin C concentrations (Table 1).

**Table 1:** Baseline characteristics of the participantsaccording to by cystatin C concentration quartiles.

Characteristics	Overall	Cystatin C (mg/L)			
		Q1 (< 0.84)	Q2 (0.84–0.97)	Q3 (0.98–1.20)	Q4 (> 1.20)
No. of participants	13,913	3461	3492	3460	3500
Age, years	65.03 (10.29)	60.47 (8.02)	63.05 (8.94)	65.69 (10.00)	70.86 (10.97)
Women (%)	8072 (58.0)	2062 (59.6)	1996 (57.2)	1961 (56.7)	2053 (58.7)
**Race (%)**					
White	10,302 (74.0)	2357 (68.1)	2646 (75.8)	2625 (75.9)	2674 (76.4)
Black	2542 (18.3)	729 (21.1)	594 (17.0)	583 (16.8)	636 (18.2)
Other	1069 (7.7)	375 (10.8)	252 (7.2)	252 (7.3)	190 (5.4)
**Household income (%), $**					
< 20,000	3543 (25.5)	751 (21.7)	707 (20.2)	875 (25.3)	1210 (34.6)
20,001–50,000	4539 (32.6)	972 (28.1)	1139 (32.6)	1174 (33.9)	1254 (35.8)
> 50,000	5831 (41.9)	1738 (50.2)	1646 (47.1)	1411 (40.8)	1036 (29.6)
**Education levels, years (%)**					
< 12	3091 (22.2)	658 (19.0)	634 (18.2)	818 (23.6)	980 (28.0)
12–15	7690 (55.3)	1805 (52.2)	1968 (56.4)	1972 (57.0)	1945 (55.6)
> 15	3133 (22.5)	998 (28.8)	890 (25.5)	670 (19.4)	575 (16.4)
BMI, kg/m^2^	28.75 (6.10)	27.69 (5.41)	28.47 (5.73)	29.17 (6.09)	29.67 (6.90)
Current smoker (%)	2235 (16.1)	566 (16.4)	544 (15.6)	601 (17.4)	524 (15.0)
Current drinker (%)	5016 (36.1)	1565 (45.2)	1423 (40.8)	1188 (34.3)	840 (24.0)
Regular exercise (%)	11,241 (80.9)	3025 (87.5)	2987 (85.5)	2813 (81.3)	2416 (69.1)
HDL-C, mg/dL	54.33 (16.15)	54.88 (16.55)	55.43 (16.35)	54.34 (16.04)	52.70 (15.51)
HbA1c, %	5.89 (1.09)	5.85 (1.18)	5.81 (1.02)	5.88 (1.06)	6.02 (1.09)
TC, mg/dL	200.86 (42.42)	200.81 (41.98)	203.86 (41.56)	203.28 (42.69)	195.53 (42.95)
HsCRP, mg/dL	4.21 (7.87)	2.77 (5.16)	3.60 (5.44)	4.22 (6.69)	6.21 (11.72)
**Prevalent health conditions**					
Pulmonary disorder (%)	1198 (8.6)	196 (5.7)	241 (6.9)	320 (9.2)	441 (12.6)
Heart disease (%)	2892 (20.8)	439 (12.7)	544 (15.6)	699 (20.2)	1210 (34.6)
Stroke (%)	939 (6.7)	120 (3.5)	171 (4.9)	225 (6.5)	423 (12.1)
Psychological problems (%)	2220 (16.0)	498 (14.4)	537 (15.4)	577 (16.7)	608 (17.4)
Hypertension (%)	7648 (55.0)	1466 (42.4)	1656 (47.4)	1947 (56.3)	2579 (73.7)
Diabetes (%)	2875 (20.7)	558 (16.1)	577 (16.5)	659 (19.0)	1081 (30.9)
CESD-8 score	1.53 (2.03)	1.48 (2.00)	1.41 (1.97)	1.50 (2.05)	1.74 (2.09)
Limitations in ADLs (%)	1052 (7.6)	200 (5.8)	202 (5.8)	252 (7.3)	398 (11.4)

Values are expressed as the mean (standard deviation) or number (percentage).

ADLs, activities of daily living; BMI, body mass index; CES-D 8 score, the 8-question Center for Epidemiologic Studies Depression Scale; high-sensitivity C-reactive protein concentration, hsCRP; HbA1c, hemoglobin A1c; HDL-C, high-density lipoprotein cholesterol; TC, total cholesterol.

### Association of cystatin C concentrations with all-cause and cause-specific mortality

**Figure 1:**
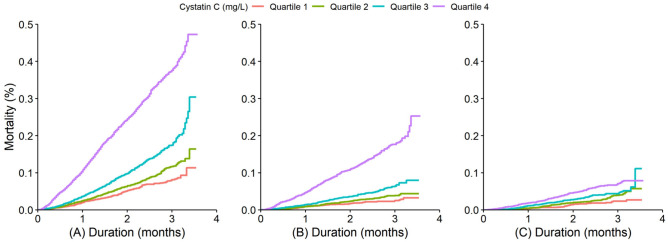
Kaplan–Meier curves for all-cause, cardiovascular and cancer mortality stratified by baseline cystatin C concentration quartiles. (**A**) Kaplan–Meier curves of all-cause mortality; (**B**) Kaplan–Meier curves of cardiovascular mortality; (**C**) Kaplan–Meier curves of cancer mortality. quartile 1 (Q1, < 0.84 mg/L), quartile 2 (Q2, 0.84–0.97 mg/L), quartile 3 (Q3, 0.98–1.20 mg/L), and quartile 4 (Q4, > 1.20 mg/L).

**Figure 2:**
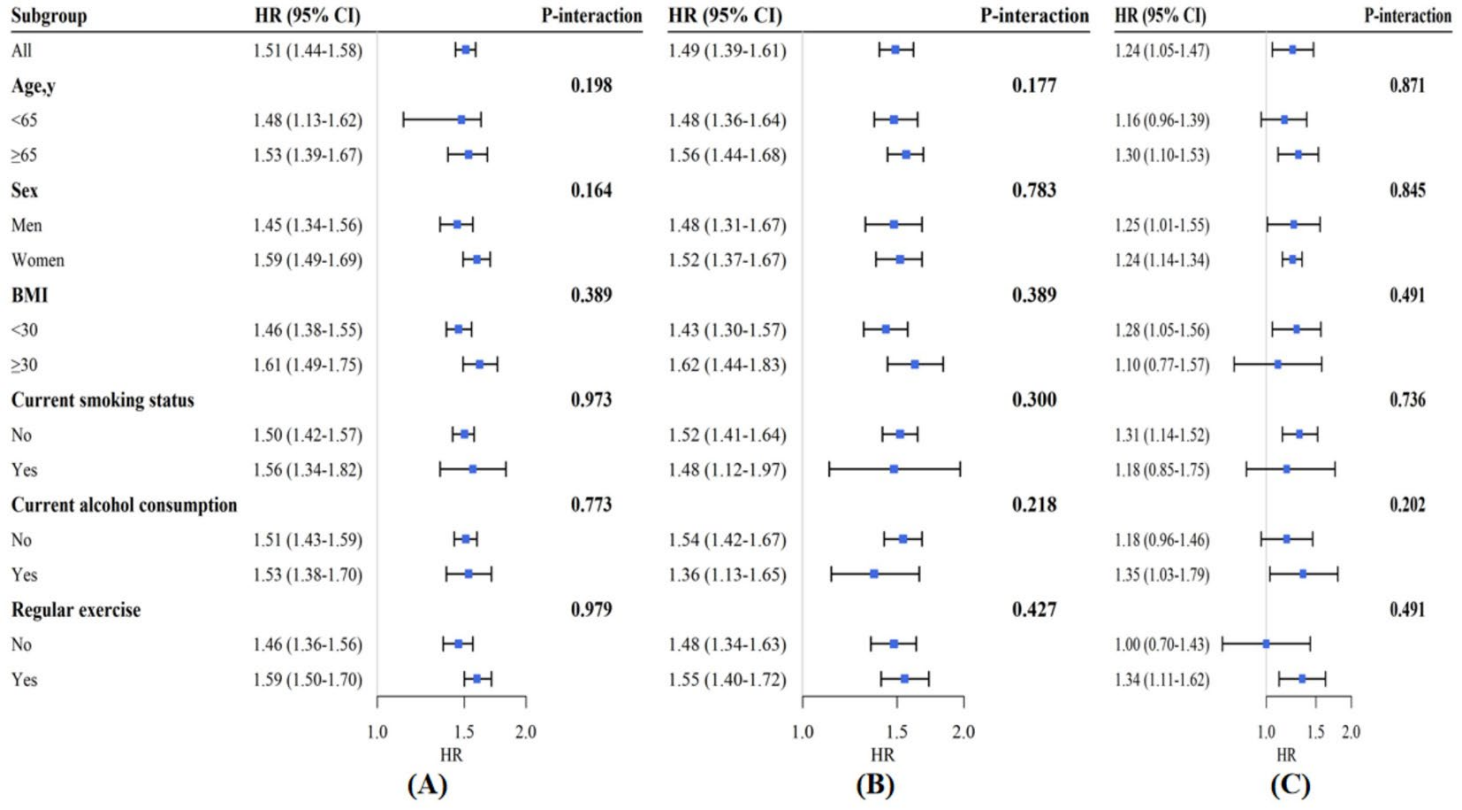
Subgroup analyses for the hazard ratio of all-cause (**A**), cardiovascular (**B**) and cancer mortality (**C**) for each 1 mg/L increase in cystatin C concentrations. Adjusted for age, sex, race, educational level, current smoking status, alcohol consumption, regular exercise, body mass index (BMI), household income, total cholesterol (TC) concentration, high-sensitivity C-reactive protein concentration, high density lipoprotein cholesterol (HDL-C) concentration, hemoglobin A1c (HbA1c) concentration, CES-D 8 score, hypertension, heart disease, stroke, cancer, diabetes, pulmonary disorders, psychiatric problems, and limitations in activities of daily living (ADLs).

During a total of 71,988 person-years of follow-up (median follow-up: 5.8 years, interquartile range 3.3–7.6 years), 1893 deaths were documented, including 714 from cardiovascular diseases and 406 from cancer. Rates of all-cause, cardiovascular and cancer mortality increased in association with increases in cystatin C concentrations assessed as quartiles (Fig. 1). The multivariable-adjusted HRs (Model 2) of all-cause, cardiovascular and cancer mortality with the lowest quartile (Q1) of cystatin C concentrations as the reference were 1.92 (1.62–2.28), 1.98 (1.48–2.65), and 1.62 (1.13–2.32), respectively, for the highest quartile (Q4) (all P for trends < 0.05) (Table 2). Additionally, evaluating the risks of all-cause, cardiovascular, and cancer mortality associated with each 1 mg/L increase in cystatin C concentrations revealed multivariable-adjusted HRs (95% CIs) of 1.51 (1.44–1.58), 1.49 (1.39–1.61), and 1.24 (1.05–1.47), respectively (Fig. 2).

**Table 2:** HRs (95% CIs) for all-cause, cardiovascular and cancer mortality according to cystatin C concentration quartiles.

Cystatin C quartiles	All-cause mortality		Cardiovascular mortality		Cancer mortality	
	Model 1^†^	Model 2^‡^	Model 1	Model 2	Model 1	Model 2
No. of participants	13,913		13,913		13,913	
Person-years at risk	71,988		71,988		71,988	
No. of events	1892		714		406	
Q1	1.00 (reference)	1.00 (reference)	1.00 (reference)	1.00 (reference)	1.00 (reference)	1.00 (reference)
Q2	1.12 (0.93–1.35)	1.11 (0.92–1.33)	1.11 (0.80–1.53)	1.06 (0.77–1.46)	1.47 (1.03–2.09)*	1.45 (1.02–2.08)*
Q3	1.35 (1.13–1.60) ***	1.21 (1.01–1.44) **	1.35 (1.00–1.83)*	1.15 (0.84–1.56)	1.54 (1.08–2.19)*	1.40 (0.98–1.99)
Q4	2.44 (2.07–2.88)***	1.92 (1.62–2.28)***	2.89 (2.18–3.84)***	1.98 (1.48–2.65)***	1.93 (1.36–2.72)***	1.62 (1.13–2.32)***
*P* for trend	< 0.001	< 0.001	< 0.001	< 0.001	< 0.001	0.078

HR: hazard ratio; CI: confidence interval.

^†^Model 1: adjusted for age and sex.

^‡^Model 2: adjusted for age, sex, race, educational level, current smoking status, alcohol consumption, regular exercise, body mass index, household income, total cholesterol concentration, high-sensitivity C-reactive protein concentration, high-density lipoprotein-cholesterol concentration, hemoglobin A1c, CES-D 8 score, hypertension, heart disease, stroke, cancer, diabetes, pulmonary disorders, psychiatric problems, and limitations in activities of daily living (ADLs).

*P < 0.05; **P < 0.01; ***P < 0.001.

### Subgroup and sensitivity analyses

We conducted subgroup analyses according to potential risk factors (Fig. 2). We observed no evidence of a significant difference for age group (≥ 65 years and < 65 years), sex (men or women), current smoking status (smoker or nonsmoker), current alcohol consumption (drinker or nondrinker), regular exercise (yes or no), or BMI (< 30 or ≥ 30 kg/m^2^) (all P for interaction > 0.05) regarding the associations of cystatin C concentrations with all-cause, cardiovascular and cancer mortality (Fig. 2). Sensitivity analyses showed no substantial change when we excluded those who participants who died in the first 2 years of follow-up (Table S1), when participants were divided into tertiles (Table S2) or quintiles (Table S3) based on cystatin C concentrations, and when we additionally adjusted for the time of measurement laboratory (Table S4).

## Discussion

In a large-scale longitudinal analysis of middle-aged and elderly individuals, this study found that elevated concentrations of cystatin C are associated with an increased risk of all-cause, cardiovascular and cancer mortality. Such associations were independent of other potential confounders, including sex, age, income, body mass index, physical activity, healthy diet, alcohol intake, smoking status, diabetes, and hypertension. Specifically, the associations of cystatin C concentrations with all-cause, cardiovascular and cancer mortality did not differ substantially when participants were stratified by sex, age, BMI, current smoking status, current alcohol consumption, and regular exercise (all P for interactions > 0.05).

Our results confirm the findings of previous studies that indicated positive associations between plasma cystatin C concentrations and the risks of all-cause and cardiovascular mortality^8,10,12,28^. Some possible explanations for the positive association of cystatin C with all-cause and cardiovascular mortality have been proposed. First, cystatin C has been suggested to be a promising measure of kidney function, while kidney dysfunction is an acknowledged risk factor for all-cause and cardiovascular mortality. Second, cystatin C has also been shown to be a marker of inflammation^29^. Furthermore, a higher cystatin C concentration was also associated with incident prediabetes^30^, hypertension^31^, coronary heart disease^32,33^, myocardial infarction^8^, and stroke^8,34^, all of which could contribute to increased mortality. With regard to cancer mortality, there is very limited evidence examining the association of cystatin C with cancer mortality^18^. Our study indicated that a higher cystatin C concentration is associated with an increased risk of cancer mortality. The result is consistent with previous studies showing a potential association between markers of CKD and a higher risk of incidence and death from overall and site-specific cancer^35–38^. Cystatin C is rarely used to measure renal function in actual practice, which may be due to other unknown or unmeasured factors contributing to this effect, and this issue should be addressed in future studies. Overall, monitoring cystatin C concentration may help identify individuals who are at higher risk of all-cause, cardiovascular and cancer mortality.

Our result is consistent with previous studies demonstrating that positive associations between cystatin C and all-cause mortality in both males and females^8,25^. In contrast, Toft and colleagues^13^ found that cystatin C concentrations were positively associated with in a female population but not in a male population. We speculate that the difference in these findings may be explained by different study populations, diverse cutoff levels, various controlled confounders, or insufficient statistical power. Our study also showed that the association of cystatin C concentrations and mortality appeared to be similar in participants aged < 65 years and those aged ≥ 65 years, consistent with previous studies^12^. Moreover, the associations of cystatin C concentrations with all-cause, cardiovascular and cancer mortality did not differ substantially when participants were stratified by BMI, current smoking status, current alcohol consumption, and regular exercise. These findings imply that cystatin C may be a useful marker for all-cause, cardiovascular and cancer mortality risk estimation, which does not differ by subgroups of sex, age, BMI, current smoking status, current alcohol consumption, and regular exercise.

## Strengths and limitations

The major strengths of the current study are the community-based, prospective design, the large sample of middle-aged and older participants, the adjustments for several identified and potential confounders, and the robust findings of the subgroup and sensitivity analyses. However, our study has several potential limitations that should be considered. First, the measurements of plasma cystatin C concentrations were only single baseline examinations, which may not accurately reflect the long-term plasma cystatin C concentrations of the study participants. Repeated plasma cystatin C measurements might reduce the variability; however, repeated longitudinal measurements of cystatin C concentrations in large-scale community-based cohort studies are impractical and expensive. Second, we cannot be confirming whether the strong association of cystatin C with the mortality is due solely to its relationship with kidney function. Cystatin C may have potential toxic effects that also help to the strength of its association with mortality^8^. Finally, although we carefully adjusted for several confounders, such as sociodemographic characteristics and lifestyle factors, the potential for residual confounding factors, such as other unmeasured or unknown covariates, likely remained.

In conclusion, this study indicates that an elevated plasma cystatin C concentration is associated with the risk of all-cause, cardiovascular and cancer mortality both men and women among the middle-aged and elderly individuals. The results demonstrate the potential value of cystatin C as a biomarker for risk prediction in middle-aged and older people.

## Supplementary Information

Supplementary Information.

## Data Availability

Publicly available datasets were analyzed in this study. This data can be found at: https://hrs.isr.umich.edu/about.
