# Supplementary material for: Association of plasma cystatin C with all-cause and cause-specific mortality among middle-aged and elderly individuals: a prospective community-based cohort study
Source: Sci Rep. 2022 Dec 23;12:22265. doi: 10.1038/s41598-022-24722-4 (PMC9789032; doi:10.1038/s41598-022-24722-4)
Supplement: Supplementary file 1 — Supplementary Information. [file 41598_2022_24722_MOESM1_ESM.docx]

**Supplement Material:**


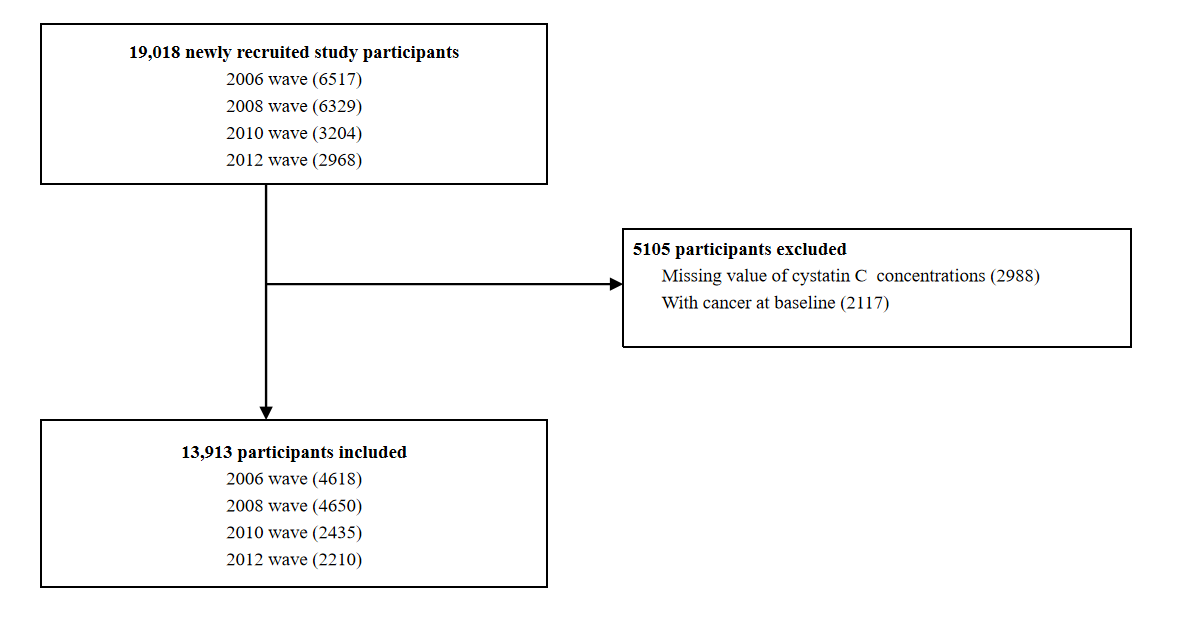


**Figure S1.** Flowchart of the participant enrolment.

**Table S1.** Multivariable hazard ratios (HR [95% CI]) for mortality by quartiles of cystatin C after excluding death in the first two years of follow-up

| HsCRP quartiles (mg/L) | All-cause mortality |  | Cardiovascular mortality |  | Cancer mortality |
| --- | --- | --- | --- | --- | --- |
| Q1 | 1.00 (reference) |  | 1.00 (reference) |  | 1.00 (reference) |
| Q2 | 1.12 (0.92-1.36) |  | 1.11 (0.79-1.55) |  | 1.43 (0.99-2.06) |
| Q3 | 1.22 (1.01-1.47) |  | 1.13 (0.82-1.56) |  | 1.44 (1.00-2.07) |
| Q4 | 1.86 (1.55-2.23) |  | 1.90 (1.39-2.58) |  | 1.55 (1.07-2.25) |
| *P* for trend | <0.001 |  | <0.001 |  | <0.001 |

**^†^** Adjusted for age, sex, race, educational level, current smoking status, alcohol consumption, regular exercise, body mass index (BMI), household income, total cholesterol (TC) concentration, high-sensitivity C-reactive protein concentration, high density lipoprotein cholesterol (HDL-C) concentration, hemoglobin A1c (HbA1c) concentration, CES-D 8 score, hypertension, heart disease, stroke, cancer, diabetes, pulmonary disorders, psychiatric problems, and limitations in activities of daily living (ADLs)

**Table S2.** Multivariable hazard ratios (HR [95% CI]) for mortality by tertiles of cystatin C

| Cystatin C tertiles (mg/L) | All-cause mortality |  | Cardiovascular mortality |  | Cancer mortality |
| --- | --- | --- | --- | --- | --- |
| Q1 (<1.14 mg/L) | 1.00 (reference) |  | 1.00 (reference) |  | 1.00 (reference) |
| Q2 (1.14-2.78 mg/L) | 1.13 (0.96-1.32) |  | 1.06 (0.80-1.39) |  | 1.51 (1.09-2.09) |
| Q3 (>2.78 mg/L) | 1.74 (1.49-2.02) |  | 1.82 (1.40-2.36) |  | 1.56 (1.13-2.15) |
| *P* for trend | <0.001 |  | <0.001 |  | 0.009 |

**^†^** Adjusted for age, sex, race, educational level, current smoking status, alcohol consumption, regular exercise, body mass index (BMI), household income, total cholesterol (TC) concentration, high-sensitivity C-reactive protein concentration, high density lipoprotein cholesterol (HDL-C) concentration, hemoglobin A1c (HbA1c) concentration, CES-D 8 score, hypertension, heart disease, stroke, cancer, diabetes, pulmonary disorders, psychiatric problems, and limitations in activities of daily living (ADLs).

**Table S3.** Multivariable hazard ratios (HR [95% CI]) for mortality by quintiles of cystatin C

| Cystatin C quintiles (mg/L) | All-cause mortality |  | Cardiovascular mortality |  | Cancer mortality |
| --- | --- | --- | --- | --- | --- |
| Q1 | 1.00 (reference) |  | 1.00 (reference) |  | 1.00 (reference) |
| Q2 | 1.01 (0.79-1.27) |  | 0.83 (0.55-1.26) |  | 1.13 (0.71-1.80) |
| Q3 | 1.21 (1.00-1.47) |  | 1.04 (0.75-1.45) |  | 1.30 (0.87-1.95) |
| Q4 | 1.25 (1.03-1.52) |  | 1.22 (0.88-1.70) |  | 1.78 (1.22-2.59) |
| Q5 | 2.08 (1.72-2.51) |  | 2.10 (1.54-2.87) |  | 1.68 (1.13-2.51) |
| *P* for trend | <0.001 |  | <0.001 |  | <0.001 |

**^†^** Adjusted for age, sex, race, educational level, current smoking status, alcohol consumption, regular exercise, body mass index (BMI), household income, total cholesterol (TC) concentration, high-sensitivity C-reactive protein concentration, high density lipoprotein cholesterol (HDL-C) concentration, hemoglobin A1c (HbA1c) concentration, CES-D 8 score, hypertension, heart disease, stroke, cancer, diabetes, pulmonary disorders, psychiatric problems, and limitations in activities of daily living (ADLs).

**Table S4.** Multivariable hazard ratios (HR [95% CI]) for mortality by quartiles of cystatin C after adjusted for measurement laboratory

| HsCRP quartiles (mg/L) | All-cause mortality |  | Cardiovascular mortality |  | Cancer mortality |
| --- | --- | --- | --- | --- | --- |
| Q1 | 1.00 (reference) |  | 1.00 (reference) |  | 1.00 (reference) |
| Q2 | 1.12 (0.93-1.35) |  | 1.06 (0.77-1.47) |  | 1.43 (0.99-2.06) |
| Q3 | 1.21 (1.01-1.45) |  | 1.15 (0.85-1.56) |  | 1.44 (1.00-2.07) |
| Q4 | 1.93 (1.62-2.29) |  | 1.98 (1.48-2.65) |  | 1.62 (1.13-2.31) |
| *P* for trend | <0.001 |  | <0.001 |  | <0.001 |

**^†^** Adjusted for age, sex, race, educational level, current smoking status, alcohol consumption, regular exercise, body mass index (BMI), household income, total cholesterol (TC) concentration, high-sensitivity C-reactive protein concentration, high density lipoprotein cholesterol (HDL-C) concentration, hemoglobin A1c (HbA1c) concentration, CES-D 8 score, hypertension, heart disease, stroke, cancer, diabetes, pulmonary disorders, psychiatric problems, limitations in activities of daily living (ADLs) , and measurement of cystatin C concentrations laboratory.
